# Biofilm formation and virulence expression by *Streptococcus mutans *are altered when grown in dual-species model

**DOI:** 10.1186/1471-2180-10-111

**Published:** 2010-04-14

**Authors:** Zezhang T Wen, David Yates, Sang-Joon Ahn, Robert A Burne

**Affiliations:** 1 Department of Oral and Craniofacial Biology and Department of Microbiology, Immunology and Parasitology, Louisiana State University Health Sciences Center, New Orleans, LA 70119, USA; 2 Department of Oral Biology, College of Dentistry, University of Florida, Box 100424, Gainesville, FL 32610, USA

## Abstract

**Background:**

Microbial cell-cell interactions in the oral flora are believed to play an integral role in the development of dental plaque and ultimately, its pathogenicity. The effects of other species of oral bacteria on biofilm formation and virulence gene expression by *Streptococcus mutans*, the primary etiologic agent of dental caries, were evaluated using a dual-species biofilm model and RealTime-PCR analysis.

**Results:**

As compared to mono-species biofilms, biofilm formation by *S. mutans *was significantly decreased when grown with *Streptococcus sanguinis*, but was modestly increased when co-cultivated with *Lactobacillus casei*. Co-cultivation with *S. mutans *significantly enhanced biofilm formation by *Streptococcus oralis *and *L. casei*, as compared to the respective mono-species biofilms. RealTime-PCR analysis showed that expression of *spaP *(for multi-functional adhesin SpaP, a surface-associated protein that *S. mutans *uses to bind to the tooth surface in the absence of sucrose), *gtfB *(for glucosyltransferase B that synthesizes α1,6-linked glucan polymers from sucrose and starch carbohydrates) and *gbpB *(for surface-associated protein GbpB, which binds to the glucan polymers) was decreased significantly when *S. mutans *were co-cultivated with *L. casei*. Similar results were also found with expression of *spaP *and *gbpB*, but not *gtfB*, when *S. mutans *was grown in biofilms with *S. oralis*. Compared to mono-species biofilms, the expression of *luxS *in *S. mutans *co-cultivated with *S. oralis *or *L. casei *was also significantly decreased. No significant differences were observed in expression of the selected genes when *S. mutans *was co-cultivated with *S. sanguinis*.

**Conclusions:**

These results suggest that the presence of specific oral bacteria differentially affects biofilm formation and virulence gene expression by *S. mutans*.

## Background

Oral biofilms are compositionally and structurally complex bacterial communities. To date, more than 750 different species or phylotypes of bacteria have been identified in mature dental plaque [1]. Microbial cell-cell interactions in the oral flora and their impact on bacterial adherence and biofilm formation are beginning to be appreciated [1-4]. Cross-feeding or metabolic cooperation is well-documented among certain bacterial species in the oral flora. Veillonellae can utilize the lactic acid produced by streptococci and *Porphyromonas gingivalis *benefits from succinate produced by *T. denticola*. Similarly, isobutyrate secreted by *P. ginivalis *stimulates the growth of *T. denticola *[2,3]. Adhesin-ligand mediated physical interactions such as those between *Streptococcus gordonii *and *P. gingivalis *may be important for secondary colonizers like *P. gingivalis *to establish and persist in the oral cavity [5]. A recent study has also provided evidence that a mutualistic effect in biofilm formation between *Actinomyces naeslundii *and *Streptococcus oralis *is facilitated by autoinducer-2 (AI-2) [6]. Intra- and inter-species interactions are believed to play a crucial role in community dynamics, contributing to the formation of plaque and, ultimately, the development of polymicrobial diseases, including caries and periodontitis [2,5]. Therefore, a better understanding of cell-cell interactions between oral pathogens and commensal bacteria, and the impact of these interactions on expression of virulence factors and pathogenicity, could lead to development of novel preventive and therapeutic strategies against dental caries and periodontitis.

As the principal etiological agent of human dental caries, *Streptococcus mutans *has developed multiple mechanisms to colonize the tooth surface and, under certain conditions, to become a numerically significant species in cariogenic biofilms [7]. The multi-functional adhesin SpaP, also called P1 and PAc1, is considered the primary factor mediating early attachment of *S. mutans *to tooth enamel in the absence of sucrose [8]. *S. mutans *also produces at least three glucosyltransferases (GtfB, -C & -D), which polymerize the glucosyl moiety from sucrose and starch carbohydrates into α1,3- and α1,6-linked glucans [7,9]. Binding to glucans by glucan binding proteins (GbpA, -B, -C and -D) and by the Gtfs facilitates bacterial adherence to tooth surfaces, inter-bacterial adhesion and accumulation of biofilms [9,10]. GtfBC&D and GbpABC&D, together with the adhesive extracellular glucans, constitute the sucrose-dependent pathway for *S. mutans *to establish on the tooth surface and are of central importance in plaque formation and development of caries [7,9-14].

Multiple regulatory networks that integrate external signals, including the cell density-dependent Com system and other two-component regulatory systems, including CiaHR, LiaSR and VicRK, with CiaH, LiaS and VicK being the sensor kinases and CiaR, LiaR and VicR the response regulators of two-component system, are required for biofilm formation [15-21]. *S. mutans *also possesses a LuxS-mediated signaling pathway that affects biofilm formation and bacteriocin production [18,22,23]. LuxS is the enzyme that catalyzes the reactions leading to the production of the AI-2 signal molecule [24]. In addition, a number of other gene products, such as BrpA (a cell surface-associated biofilm regulatory protein), have also been shown to play critical roles in environmental stress responses and biofilm development by *S. mutans *[25,26]. While much effort has been devoted to understanding the molecular mechanisms of adherence, biofilm development and virulence gene expression by *S. mutans *in pure cultures, there are large gaps in our knowledge of how this cariogenic bacterium behaves in response to inter-generic interactions with bacteria commonly found in the supragingival plaque.

In this study, we developed a dual-species *in vitro *model to examine the impact of co-cultivation of *S. mutans *with *S. oralis *or *S. sanguinis*, two primary colonizers and members of the normal flora, or with *Lactobacillus casei*, a bacterium frequently isolated from carious sites, on biofilm formation by these bacteria and expression of known virulence factors of *S. mutans*. Data presented here suggest that growth in dual-species impacts surface biomass accumulation by some of the bacterial species analyzed, as compared to the respective mono-species biofilms and that the expression of known virulence factors by *S. mutans *can be differentially modulated by growth with other bacteria commonly found in dental plaque. Such interactions may influence the formation, architecture and pathogenic potential of human dental plaque.

## Methods

### Bacterial strains and growth conditions

*S. mutans *UA159, *S. oralis *SK92 and *S. sanguinis *SK150 were maintained in Brain Heart Infusion (BHI, Becton, Dickinson and Company, MD), and *L. casei *4646 was maintained in Lactobacillus MRS (Difco Laboratories, MI). For biofilm formation, all cultures were grown on semi-defined biofilm medium (BM) [27] with 18 mM glucose and 2 mM sucrose as the supplemental carbohydrate sources (BMGS) [18,26]. Biofilms were grown in a 5% CO_2_-aerobic atmosphere at 37°C. For growth studies using a Bioscreen C (Oy Growth Curves AB Ltd, Finland), cultures were grown at 37°C aerobically and the optical densities were monitored every 30 minutes, with shaking for 10 seconds before measurement [28].

### Growth of dual-species biofilms

Sterile glass slides were used as substratum and biofilms were grown by following a protocol described previously [25,26]. Briefly, overnight broth cultures were transferred by 1:50 dilutions into fresh, pre-warmed, broth medium (BHI for streptococci and MRS for lactobacillus) and were allowed to grow until mid-exponential phase (OD_600 nm _≅ 0.5) before transfer to BMGS for biofilm formation. For mono-species biofilms, 450 μl of the individual cultures was added to the culture tube, and for two-species biofilms, 450 μl of each culture was used as inoculum. The biofilms grown on the glass slides that were deposited in 50 ml Falcon tubes were aseptically transferred daily to fresh BMGS. After four days, the biofilms were scratched off with a sterile spatula and suspended in 7.5 ml of 10 mM potassium phosphate buffer, pH 7.0. To de-chain and separate the cells, the biofilms were sonicated using a Sonic Dismembrator (model 100, Fisher Scientific, Idaho) at energy level 3 for 25 seconds, twice, with 2 minutes on ice between treatments. To determine the total number of viable bacterial cells (colony-forming units, CFU), 100 μl from dispersed, four-day biofilms was serially diluted in potassium phosphate buffer, 10 mM, pH 7.0, and plated in triplicate on BHI agar plates.

### RNA extraction

Immediately after sampling for plating, bacterial cells were treated with RNAProtect (Qiagen Inc., CA) as recommended by the supplier. The cells were then pelleted by centrifugation and total RNA extractions were performed using a hot phenol method [18,26]. To remove all DNA, the purified RNAs were treated with DNAse I (Ambion, Inc., TX) and RNA was retrieved with the Qiagen RNeasy purification kit, including an additional on-column DNAse I treatment with RNase-free DNase I.

### RealTime-PCR

For RealTime-PCR, gene-specific primers were designed using the DNA mfold program http://mfold.bioinfo.rpi.edu/cgi-bin/dna-form1.cgi and Beacon Designer 3.0 (PREMIER Biosoft International, Palo Alto, CA) using the following criteria: primer length 20-22 nucleotides, T_m _≥ 60°C with 50 mM NaCl and 3 mM MgCl_2_, and the expected length of PCR products 85-150 bp (Table 1). For RealTime-PCR, cDNA was generated with gene-specific primers using SuperScript III First Strand Synthesis Kit (InVitorgen, CA) by following the supplier's recommendations. For validation assays, iScript Reverse Transcriptase was also used to generate cDNA templates with random nanomers as primers (Bio-Rad laboratories, CA). RealTime-PCR was conducted using the iCycler iQ real time PCR detection system (Bio-Rad Laboratories), with controls and standards as described previously [20,26].

**Table 1:** Primers used for RealTime-PCR

Primer	DNA sequence (5' → 3')	Application	Amplicon
spaP-Fw	TCCGCTTATACAGGTCAAGTTG	*spaP *fragment	121 bp
spaP-Rv	GAGAAGCTACTGATAGAAGGGC		
gtfB-Fw	AGCAATGCAGCCATCTACAAAT	*gtfB *fragment	98 bp
gtfB-Rv	ACGAACTTTGCCGTTATTGTCA		
gbpB-Fw	CGTGTTTCGGCTATTCGTGAAG	*gbpB *fragment	108 bp
gbpB-Rv	TGCTGCTTGATTTTCTTGTTGC		
luxS-Fw	ACTGTTCCCCTTTTGGCTGTC	*luxS *fragment	93 bp
luxS-Rv	AACTTGCTTTGATGACTGTGGC		
brpA-Fw	CGTGAGGTCATCAGCAAGGTC	*brpA *fragment	148 bp
brpA-Rv	CGCTGTACCCCAAAAGTTTAGG		
ldh-Fw	TTGGCGACGCTCTTGATCTTAG	*ldh *fragment	92 bp
ldh-Rv	GTCAGCATCCGCACAGTCTTC		

### Data analysis

The mRNA copy number of selected virulence factors was determined per μg of total RNA. When grown in the dual-species model, the values were further normalized to relative numbers of *S. mutans *by multiplying the copy number by the ratio of *S. mutans *CFU to the total CFU in the mixed-species biofilms. The resulting data were expressed as copy number per μg of *S. mutans *total RNA. Statistical analysis was carried out using the non-parametric Kruskal-Wallis test and *t*-test.

## Results and Discussion

### Establishment of a suitable biofilm model for the reliable monitoring of gene expression in *S. mutans*

Glass slides can be used very effectively to cultivate biofilms of oral bacteria [26,29]. As compared to tooth enamel model systems, e.g. hydroxylapatite disks, glass slides are easier to handle, stable and non-reactive. By daily transfer to fresh medium, bacteria on glass surfaces continue to accumulate and generate sufficient biofilms after 3-4 days for multiple experiments [29], including whole genome transcriptional profiling [26].

For measurement of the expression levels of selected virulence factors by *S. mutans*, total RNA was extracted from mono- and dual-species biofilms and RealTime-PCR reactions were carried out using gene-specific primers (Table 1). To confirm that no genomic DNAs left in the RNA preps, cDNA synthesis reactions that received no reverse transcriptase were used as controls and results of RealTime-PCR using gene-specific primers (Table 1) showed that none of the RNA preps used in this study had any significant genomic DNA contamination. To verify that the primers did not amplify non-*S. mutans *genes under the conditions tested, total RNA of *S. oralis, S. sanguinis *and *L. casei*, either alone or in mixtures with known quantities of *S. mutans *RNA, were used as a template for reverse transcription and RealTime-PCR. No cDNA was detected when *S. oralis, S. sanguinis *or *L. casei *total RNA alone was used as a template with primers for *spaP*, *gtfB, gbpB, luxS*, and *brpA*, as well as the *ldh *gene encoding lactate dehydrogenase) (data not shown). Melting curves consistently presented unique amplification products for every amplicon tested. Results also demonstrated that the presence of RNA from non-mutans streptococci or from lactobacilli had no significant effect on the efficiency of amplification of *S. mutans *specific products. Specifically, quantification of the respective genes in mixed RNA samples yielded results that were proportional to the amount of *S. mutans *RNA used in the reactions (data not shown). Similar results were also obtained with genomic DNA from the respective strains as templates (data not shown).

The choice of appropriate controls for this study was carefully considered. Ribosomal RNA is the most commonly used reference in single species transcriptional analysis, and has often been used as a control in Northern analysis of *S. mutans *RNA [18,30]. However, use of ribosomal RNAs could be misleading when it is used for analysis of gene expression in mixed-species biofilms, especially when closely related species are present in the consortium. Specifically, during calibration of the methods, cross-reactions between rRNA of different bacterial sources were noted, as shown by multiple peaks in the melting curves in the RealTime-PCR reactions (data not shown). Therefore, rather than use rRNA total viable counts (CFU) were used to normalize the RealTime-PCR data. Brief sonication was used to disperse the biofilms. When plated on BHI agar plates, the distinctive colony morphology of *S. mutans *(flat, opaque, dry colonies with rough surface) versus *S. oralis *(small, flat and smooth colonies), *S. sanguinis *and *L. casei *(both forming small, wet, convex colonies with shiny and smooth surfaces) made it easy to distinguish *S. mutans *from the other streptococci and *L. casei*. For *S. mutans-L. casei *dual-species biofilms, an erythromycin resistant *L. casei *strain (Browngardt and Burne, unpublished data) was also used in dual-species biofilms, and BHI agar plates containing erythromycin (5 μg ml^-1^) were used for viable counts of *L. casei*. The results were similar to those when BHI agar plates were used (data not shown).

**Figure 1:**
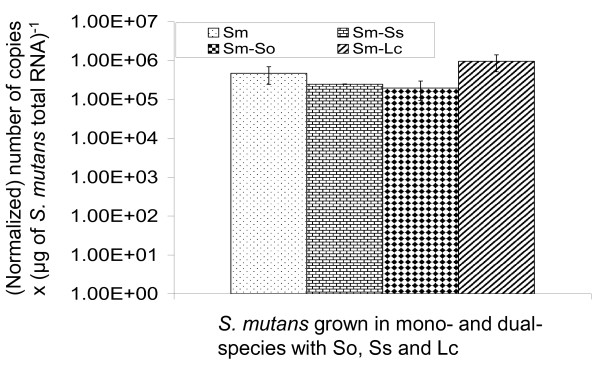
**RealTime-PCR analysis of *ldh *gene as an internal control**. Data presented here were generated from at least four separate sets of biofilm cultures and RealTime-PCR was carried out in triplicate and was repeated at least once. These data were normalized and further analyzed using a non-parametric Kruskal-Wallis Test and student *t*-test. The bar graphs represent the average (± standard deviation in error bars) of normalized copy numbers × (μg *S. mutans *total RNA)^-1^. No significant differences were observed between *S. mutans *grown in mono-species and those grown in dual-species biofilms. Abbreviations: Sm, *S. mutans*; Ss, *S. sanguinis*; So, *S. oralis*; Lc, *L. casei*, with Sm-Ss, Sm-So and Sm-Lc indicating dual-species biofilm of the selected bacteria.

The lactate dehydrogenase gene *ldh *of *S. mutans *has been reported to be constitutively expressed [31] (Wen and Burne, unpublished data), so we also examined whether this gene may serve as a suitable reference. No cross-reactions were detected between primers of *S. mutans ldh *and genes of other bacteria (data not shown). As expected, no significant difference in expression of *ldh *was observed between *S. mutans *grown in mono-species biofilms and those in dual-species biofilms, following proper normalization to CFU (Figure 1). Similar results were obtained when random primers were used to generate cDNA template instead of *ldh*-specific primers. These results add additional support to the finding that RealTime-PCR with normalization to CFU is a reliable approach for assessment of gene regulation in *S. mutans *growing in this mixed-species biofilm model.

### *S. mutans *enhances biofilm formation by *S. oralis *and *L. casei *in dual-species model

**Figure 2:**
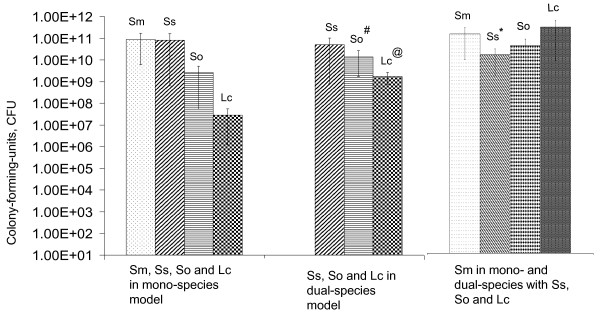
**Biofilm formation in mono- and dual-species model**. Data presented here were generated from more than ten independent sets of experiments and were further analyzed using a non-parametric Kruskal-Wallis Test and student *t*-test. This graph shows the average (± standard deviation, in error bars) of CFU in biofilms formed by *S. mutans *and the other oral bacteria tested when grown in the mono- and dual-species models with *S. mutans*. A *, # and @ indicates significant difference at *P *< 0.05, 0.01 and 0.001, respectively, when compared to those grown in mono-species biofilms. All abbreviations are the same as in Figure 1.

When grown on glass slides, *S. mutans *accumulated an average of 8.8 × 10^10 ^CFU after 4 days (Figure 2). *S. sanguinis *also formed biofilms efficiently on glass surfaces, averaging 8.2 × 10^10 ^CFU after 4 days. When these two bacteria were grown in the dual-species model, the level of *S. mutans *decreased by more than 8-fold (*P *< 0.05), yielding an average of 1.0 × 10^10 ^CFU, while *S. sanguinis *accumulated to 5.1 × 10^10 ^CFU. *S. oralis *displayed a relatively poor capacity to form biofilms when grown alone, averaging 2.6 × 10^9 ^CFU after 4 days. When grown in dual-species with *S. mutans*, however, the number of *S. oralis *in the biofilms increased to an average of 1.4 × 10^10 ^CFU (*P *< 0.01). On the other hand, biofilm formation by *S. mutans *was decreased when grown together with *S. oralis*, although the difference between mono-species and dual-species was not statistically significant. *L. casei *alone formed biofilms poorly, accumulating only 2.9 × 10^7 ^CFU after 4 days. However, the capacity of *L. casei *to form biofilms was enhanced significantly (*P *< 0.001) when co-cultivated with *S. mutans*, resulting in an increase of more than 60-fold to an average of 1.7 × 10^9 ^CFU after 4 days. Notably, when *S. mutans *was cultivated in dual-species biofilms with *L. casei*, the organisms accumulated in about 2-fold greater numbers than when *S. mutans *was grown alone, averaging 1.8 × 10^11 ^CFU.

Various bacterial cell-cell interactions may exist when growing in dual-species biofilms, including competition for binding sites and nutrients available. In this study, the same amount of inoculum was used in mono- and dual-species biofilms. For bacteria that grow similarly well in mono-species model, slight decreases will be expected for both constituents when grown in dual-species biofilms. The observed decreases in population of both *S. mutans *and *S. sanguinis *when they were cultivated together (Figure 2), as compared to the respective mono-species biofilms, could be at least in part attributed to competition for binding sites. Both *S. sanguinis *and *S. oralis *grew well in BMGS broth, with a doubling time of 86.5 (± 2.7) and 80 (± 6.1) minutes, respectively, whereas *S. mutans *took 134.7 (± 11.6) minutes to double its optical density. These results suggest that *S. sanguinis *and *S. oralis *should possess advantages over *S. mutans *for available nutrients when grown in a mixed-species consortium. Disadvantages in nutrient competition could certainly affect the capacity of *S. mutans *to accumulate on the glass surfaces, contributing to the observed decreases in biofilm formation when grown together with *S. sanguinis *or *S. oralis *(Figure 2). *S. sanguinis *is also known to produce hydrogen peroxide, which can inhibit the growth of *S. mutans *[4,32], although such an impact on *S. mutans *growth was shown to be limited when the organisms were inoculated simultaneously [32], as they were in this study.

*L. casei *did not grow well in BMGS broth, yielding an average of 4.7 × 10^7 ^CFU ml^-1 ^after 24 hours, as compared to 6.0 × 10^8 ^CFU ml^-1 ^for *S. mutans*. Poor growth could certainly contribute to poor biofilm formation by this bacterium. As was observed with dual-species biofilms, however, co-cultivation of *L. casei *and *S. mutans *planktonically in BMGS broth also increased *S. mutans *CFU by more than 3-fold, with an average CFU of 2.3 × 10^9 ^ml^-1^, although the numbers of *L. casei *remained similar to those in mono-species cultures (data not shown). The mixed-species broth cultures also had a slightly decreased doubling time (121.4 ± 8.8 minutes), as compared to *S. mutans *(134.7 ± 11.8 minutes) and *L. casei *(240 ± 24 minutes) in mono-species planktonic cultures. BHI, and especially MRS, yielded much better growth of *L. casei *than BMGS, although no major differences were observed in biofilm formation by *L. casei *when grown in BHI or MRS (data not shown).

Oral lactobacilli, such as *L. casei*, are a group of acid tolerant bacteria that are commonly isolated in relatively significant proportions from cariogenic dental plaque [33-36]. However, the ability of lactobacilli to adhere to the tooth surface was known to be poor [36]. Results presented here also suggest that *L. casei *alone does not form biofilms on glass surfaces very effectively, but biofilm formation by this bacterium can be dramatically improved when mixed with *S. mutans*. *S. mutans *produces at least three Gtf enzymes [7] that produce high molecular weight glucans that promote bacterial adhesion and biofilm accumulation. Recent studies have shown that these enzymes, especially GtfB, are capable of directly binding to *L. casei *and other oral bacteria [37]. Adsorption of Gtfs to the surface of non-mutans streptococci can promote adhesion of the bacteria via glucan-mediated pathways [37]. Thus, the Gtf enzymes of *S. mutans *and the adhesive glucans likely contribute to the enhanced biofilm formation by *L. casei*, and probably *S. oralis*, when grown in mixed-species biofilms with *S. mutans*. Notably, enhanced biofilm formation by *Lactobacillus plantarum *and *Lactobacillus rhamnosus *was noted in a mucin-based medium [38], so the presence of polysaccharides may have a general ability to promote biofilm formation by lactobacilli. However, the actual mechanism for the enhancement of *L. casei *levels in biofilms with *S. mutans *requires further investigation.

While the close association of *L. casei *and *S. mutans *in carious sites is well documented, little information is available concerning the interaction between these two bacteria with respect to *S. mutans *biofilm formation and its cariogenicity. While co-cultivation with *S. mutans *significantly enhanced biofilm formation by *L. casei*, the sessile population of *S. mutans *was also found to be increased by more than 2-fold in dual species model with *L. casei *(Figure 2), which is contrary to what was observed with the other bacteria studied. While the exact nature and the underlying mechanism await further investigation, the interaction observed between *S. mutans *and *L. casei *may partly explain the prevalence and the close association of these two bacteria in cariogenic plaque.

### Expression of genes critical to cariogenicity of *S. mutans *can be altered when grown in mixed-species biofilms

**Figure 3:**
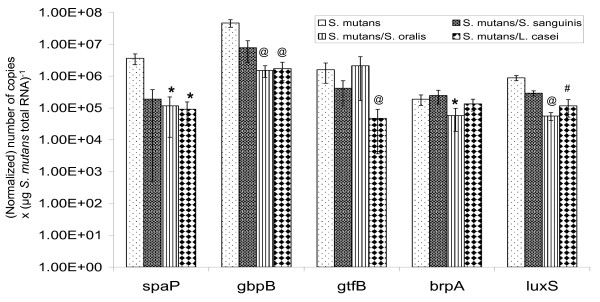
**RealTime-PCR analysis of selected genes**. RealTime-PCR for specific genes was carried out in triplicate and repeated at least once. Data presented here were generated from at least four independent sets of experiments. These data were normalized and further analyzed using a non-parametric Kruskal-Wallis Test and student *t*-test. The bar graphs represent the average (± standard deviation in error bars) of copy numbers × (μg *S. mutans *total RNA)^-1^, with *, # and @ illustrating statistical differences at *P *< 0.05, 0.01, and 0.001, respectively, when compared to the respective genes in mono-species biofilms.

RealTime-PCR was used to analyze the expression of several genes that have critical roles in bacterial adherence and biofilm accumulation by *S. mutans *[7-10], including *spaP*, *gtfB *and *gbpB*. As shown in Figure 3, slight decreases were observed in expression of *spaP, gtfB *and *gbpB *by *S. mutans *when grown in dual-species with *S. sanguinis *as compared to those in mono-species biofilms, although the differences were not statistically significant. When grown in dual-species with *L. casei*, however, expression of *spaP*, *gbpB *and *gtfB *by *S. mutans *was decreased by as much as 40-fold, at a significance level of *P *< 0.05 for *spaP *and *P *< 0.001 for *gtfB *and *gbpB*, respectively, as compared to cells in mono-species biofilms. The expression of *spaP *(*P *< 0.05) and *gbpB *(*P *< 0.001), but not *gtfB*, was also lower by more than 30-fold in *S. mutans *when grown with *S. oralis*. As compared to mono-species biofilms, expression of *luxS *was decreased by more than 7-fold in cells grown with *L. casei *(*P *< 0.001) and by more than 15-fold in cells with *S. oralis *(*P *< 0.001), but again no significant differences were observed when *S. mutans *was grown with *S. sanguinis*. Expression of *brpA *was decreased by more than 3-fold (*P *< 0.05) in cells grown with *S. oralis*, but no major differences were observed when grown with *S. sanguinis *and *L. casei*. As a control, the expression of the *ldh *gene, a constitutively expressed gene (Wen and Burne, unpublished data) [4], was also analyzed and no significant differences were observed between *S. mutans *grown in single-species and those grown in dual-species (Figure 1).

Glucosyltransferases and glucan-binding proteins of *S. mutans *are known to be differentially expressed in response to environmental conditions, such as carbohydrate source and availability, pH and growth of the bacteria on surfaces [9,39-41]. Results presented here further demonstrate that the level of expression of these known virulence attributes can be altered when *S. mutans *is grown in dual-species biofilms and that the effect varies as a function of the species of bacteria in the biofilms. Among the three different bacterial species analyzed, the most significant effect on the expression of the selected genes was seen with *L. casei*, followed by *S. oralis*. No significant effect was observed with *S. sanguinis *in expression of either *spaP, gtfB *or *gbpB*. As described above, nutrient availability, especially when grown together with faster growing microorganisms, such as *S. oralis*, could have an impact on gene expression in *S. mutans*, and consequently affect biofilm formation [42].

*L. casei*, as a frequent isolate from the cariogenic plaque, is also known for its high capacity for acid production from carbohydrates. When grown on BMGS in mono-species cultures, *S. mutans *overnight (24-hour) cultures had an average pH of 5.75 (± 0.28). As expected, the pH was decreased slightly when grown in dual species with *L. casei*, averaging 5.63(± 0.20). Similar results were also observed when *S. mutans *was grown together with *S. oralis*, with an average pH measured at 5.69 (± 0.08). In contrast, however, the pH of overnight cultures of *S. mutans *co-cultured with *S. sanguinis *was 5.95(± 0.03). Environmental pH has been shown to influence the expression of some of the genes selected [39]. Although not necessarily fully reflective of what occurs in sessile populations, the decreases in culture pH suggest that *S. mutans *may endure a more significant acid challenge when grown in dual-species with *L. casei *as well as *S. oralis *and that such decreases could at least in part contribute to the down-regulation of the selected genes in *S. mutans *grown in dual-species with *L. casei *and *S. oralis*.

Many bacteria produce autoinducer 2 through LuxS enzymes [43]. Recent studies of the oral flora have yielded evidence that AI-2 mediated signaling in the flora may play an important role in inter-species communication, affecting plaque formation and, ultimately, development of oral diseases [6,23,44,45]. Neither *S. oralis *nor *A. naeslundii *alone were found to form good biofilms, but growth in the two-species model resulted in abundant mutualistic growth [46]. AI-2 of *S. oralis *was recently found to be critical for such a mutualistic interaction [6]. Below and above the optimal concentration, mutualistic biofilm growth was suppressed. In *S. mutans*, LuxS was shown to be involved in biofilm formation and to affect the structure of biofilms [18,22,23], although its role in regulation of factors critical to bacterial adherence and biofilm formation is somewhat controversial. As shown previously, LuxS-deficiency significantly decreased *brpA *expression, but no major differences were seen between wild-type and the LuxS-deficient mutants in expression of *gtfBC, gbpB *or *spaP *[18]. Similar results were also obtained by DNA microarray analysis in both planktonic [47] (Wen et al., unpublished data) and sessile populations (Wen et al., unpublished data). In a study using RealTime-PCR, however, Yoshida et al. [23] reported that transcription of *gtfB *and *gtfC*, but not *gtfD*, was up-regulated in response to LuxS-deficiency. Like *S. mutans *and *S. oralis*, both *S. sanguinis *http://www.oralgen.lanl.gov and *L. casei *(Wen and Burne, unpublished data) possess LuxS. It remains unclear, however, whether LuxS in these bacteria is in fact involved in cell-cell communication. Nevertheless, down regulation of *luxS *expression in *S. mutans *when grown in dual-species with *L. casei *and *S. oralis *would likely affect the absolute concentration of AI-2 in the biofilms. Studies are ongoing to determine whether AI-2 signaling is functional between these bacterial species and whether alterations in *luxS *expression does in fact affect the expression of known virulence factors by *S. mutans *in mixed-species biofilms.

It is well established that GtfB and GbpB are critical components of the sucrose-dependent pathway in *S. mutans *biofilm formation and cariogenicity. In the presence of sucrose, GtfB synthesizes copious α1,3-linked, water insoluble glucan polymers. Then, surface-associated glucan-binding protein GbpB and others bind to these polymers, facilitating intercellular adherence and biofilm accumulation by *S. mutans*. It would be expected that down-regulation of GtfB and GbpB would result in less biofilm formation. Surprisingly, our *S. mutans-L. casei *dual-species data showed that *S. mutans *accumulated more than 2-fold more biofilms while the expression of *gtfB *and *gbpB *was decreased. One possible explanation is that down regulation of GtfB and GbpB (and probably some other members of the Gtfs and Gbps) when grown together with *L. casei *altered the balance of glucans to glucan-binding proteins ratio or altered the glucan structure in a way that altered biofilm architecture. In fact, similar observations have also been reported recently by us and some other groups [11,12,48]. In particular, deficiency of trigger factor (RopA) in *S. mutans *reduced production of GtfB and -D as revealed by Western blotting, but the *ropA*-mutant formed more than 50% more biofilms than the parental strain when sucrose was provided as the supplemental carbohydrate source [48]. During characterization of GbpA of *S. mutans*, the Banas group showed that strains deficient in GbpA were more adherent *in vitro *and more cariogenic *in vivo *than the parental strain [11,12]. As compared to the biofilms by the parent strain, which were composed of big cellular clusters with large gaps in between, the biofilms formed by the *gbpA*^- ^mutant were relatively small, but more compact and more evenly distributed. Interestingly, GbpA-deficiency was later found to increase the frequency of recombination between the tandemly arranged, highly homologous *gtfB *and *gtfC *genes, resulting in a dramatic decrease in production of water-insoluble glucans. Additional experiments that probe the basis for altered *gtf *and *gbp *expression, coupled with measurements of Gtf and Gbp protein and activity and glucan structure will be needed to shed light on the basis for the observations.

## Conclusions

*In vitro *dual-species biofilm model and RealTime-PCR analysis showed that biofilm formation and virulence expression by *S. mutans *could be altered in response to the presence of other oral bacterial species. Effort is currently directed to further investigation of the underlying mechanism of the altered expression of selected genes and the impact of such alterations on biofilm formation by *S. mutans*. Considering the frequent association of *L. casei *and *S. mutans *in carious sites and their role in caries development, information yielded from these studies could be used to formulate novel strategies against human dental caries.

## List of abbreviations

AI-2: autoinducer 2; GtfB, -C & -D: glucosyltransferase B, C & D; GbpA, -B, -C, & -D: glucan-binding protein A, B, C & D; CFU: colony-forming-unities; BHI: brain heart infusion; MRS: lactobacilli MRS medium; SpaP: adhesin P1; BrpA: biofilm regulatory protein A; *ldh*: lactate dehydrogenase gene; BM: biofilm medium; BMG: BM plus glucose; BMS: BM plus sucrose; BMGS: BM plus glucose and sucrose.

## Authors' contributions

ZTW conceived the study, designed and implemented most of the experiments, and drafted the manuscript; DY carried out most of the biofilm assays and RealTime-PCR analysis; SJA was involved in parts of experimental design and data analysis; RAB participated the experimental design and data analysis and revised critically the manuscript. All authors have read and approved the manuscript.
